# Clinical outcomes of limited repair and conservative approaches in older patients with acute type A aortic dissection

**DOI:** 10.1186/s13019-022-01819-5

**Published:** 2022-04-15

**Authors:** Yasumi Maze, Toshiya Tokui, Masahiko Murakami, Bun Nakamura, Ryosai Inoue, Reina Hirano, Koji Hirano

**Affiliations:** Department of Thoracic and Cardiovascular Surgery, Ise Red Cross Hospital, 1-471-2 Funae, Ise, Mie 516-8512 Japan

**Keywords:** Acute aortic dissection, Surgical outcome, Older patients, Primary entry

## Abstract

**Background:**

Surgical indication and the selection of surgical procedures for acute type A aortic dissection in older patients are controversial; therefore, we aimed to examine the surgical outcomes of acute type A aortic dissection in older patients.

**Methods:**

From January 2012 through December 2019, 174 patients underwent surgical repair for acute type A aortic dissection. We compared the surgical outcomes between the older (≥ 80 years old) and below-80 (≤ 79 years old) age groups. Additionally, we compared the outcomes between the surgical and conservative treatment groups.

**Results:**

The primary entry was found in the ascending aorta in 51.6% and 32.8% of the older and below-80 groups, respectively (*p* = 0.049). Ascending or hemiarch replacement was performed in all older group patients and 57.3% of the below-80 group patients (total arch replacement was performed in the remaining 42.7%; *p* < 0.001). Hospital mortality rates were similar in both groups. The significant risk factors for hospital mortality were age, preoperative intubation, cardiopulmonary bypass time, and postoperative stroke. The 5-year survival rates were 48.4% ± 10.3% (older group) and 86.7% ± 2.9% (below-80 group; *p* < 0.001). The rates of freedom from aortic events at 5 years were 86.9% ± 8.7% (older group) and 86.5% ± 3.9% (below-80 group; *p* = 0.771). The 5-year survival rate of the conservative treatment subgroup was 19.2% ± 8.0% in the older group, which was not significantly different from that of the surgical treatment subgroup (*p* = 0.103).

**Conclusion:**

The surgical approach did not achieve a significant survival advantage over conservative treatment and may not always be a reasonable treatment of choice for older patients.

## Background

Several problems remain despite recent improvements in the surgical outcomes of acute type A aortic dissection. In the aging society, surgical indications and surgical procedure selection are important issues for treating conditions such as acute type A aortic dissection in older patients. Surgical treatment is indicated for treating acute type A aortic dissection in both older (≥ 80-year-old) and below-80-year-old patients [1–3]. However, older patients are prone to dementia and are bedridden after surgery, thereby increasing the burden on their families [8–10]. Therefore, older patients should carefully decide on the surgical indication [4] and select a partial arch replacement or hemiarch replacement surgical procedure [5, 6]. Similar surgical outcomes have been reported in older and younger patients [7].

For the past few years, we have been selecting ascending aortic replacement (including hemiarch replacement) as the surgical procedure for acute type A aortic dissection in patients aged ≥ 80 years, regardless of the primary entry site. We aimed to examine the adequacy of the strategy used by us in this study.

## Methods

All surgeries and data collection were performed at Ise Red Cross Hospital, Ise, Japan. Clinical outcome data were obtained from the hospital’s patient records or from the patient’s family doctor.

From January 2012 to December 2019, 174 patients underwent surgical repair for acute type A aortic dissection at our institution. We divided these patients into the older (*n* = 31, ≥ 80 years old) and below-80 (*n* = 143, ≤ 79 years old) groups and compared their surgical results. Furthermore, patients with acute aortic dissection who chose conservative treatment because of comorbidities or refusal of surgical treatment were also divided into the older and below-80 groups. Within each age group, we compared the outcomes between the surgical and conservative treatment groups. The institutional review board approved the present study, and all participants provided informed consent.

### Operative techniques

The operation was performed using median sternotomy in all patients. Arterial cannulation sites (femoral artery alone or combined with right axillary artery) were determined according to the patient’s status, preoperative organ malperfusion, and surgeon’s preference. A two-stage venous cannula was inserted into the right atrium, and cardiopulmonary bypass (CPB) was established. Systemic cooling to 25 °C in the pharynx was performed. The left ventricle was vented through the right superior pulmonary vein. After the distal ascending aorta was clamped, the ascending aorta was opened, and cold cardioplegia was delivered directly into the coronary ostia. Subsequent myocardial protection was performed via retrograde infusion. A hypothermic arrest was obtained at pharynx temperatures less than 25 °C, and then the aortic arch was opened and assessed. Cerebral perfusion was achieved by antegrade selective cannulation of the orifices of all arch branches. However, when the right axillary artery was used as a cannulation site for CPB, the right axillary artery cannula was substituted for antegrade cerebral perfusion and the proximal part of the brachiocephalic artery was clamped.

An open distal anastomosis was performed regularly under moderate hypothermic circulatory arrest (25 °C) and antegrade selective cerebral perfusion. The extent of graft replacement was decided as follows: in the older group, ascending or hemiarch replacement was performed regardless of the primary entry site. In the below-80 group, ascending or hemiarch replacement was selected if the primary entry was located on the ascending aorta or the lesser curvature of the aortic arch and total arch replacement (TAR) was performed if the primary entry was located beyond the left subclavian artery or the greater curvature of the aorta. In patients undergoing TAR, all distal anastomoses were performed using either a conventional elephant trunk (ET) or frozen elephant trunk (FET) [11], depending on the age and patient condition.

In patients undergoing ascending or hemiarch replacement, reinforcement of the distal anastomosis was performed by improving the conventional adventitial inversion technique [12]. Briefly, after BioGlue® was applied to obliterate the false lumen, the redundant adventitia was inverted into the aortic lumen, and the Teflon felt that TachoSil® (fibrinogen/thrombin-based collagen fleece) was attached to the outer wall of the aorta and tacked to the luminal surface of the intima using polypropylene mattress sutures. As described above, an anastomotic site of the aorta was created.

After the distal anastomosis, whole-body circulation was resumed through the branch of the prosthesis, and the patient was fully rewarmed to 35 °C. The ascending aorta was transected at a level of 1–2 cm distal to the sinotubular junction. Proximal reinforcement and anastomosis were performed using the modified adventitial inversion technique as described above.

A single-branch prosthesis (J Shield Neo, Japan Lifeline Co. Ltd., Tokyo, Japan) was used in the ascending aortic or hemiarch replacement, and a four-branch prosthesis (J Shield Neo; Japan Lifeline Co. Ltd., Tokyo, Japan) was used in TAR. FET prosthesis (J graft Frozenix®, Japan Lifeline Co. Ltd., Tokyo, Japan) was used in the FET technique.

### Definitions

*Preoperative shock* was defined as systolic blood pressure < 80 mmHg, *cardiac tamponade* was defined as a shock caused by the cardiac effusion observed on a preoperative echocardiogram, and *malperfusion* was defined as a symptom indicating the disruption of blood flow to the end-organ systems (classified as a central nervous system, coronary, viscera, or extremities). The central nervous system disorders caused by malperfusion were classified as transient or persistent according to the duration of the clinical presentation. *Stroke* was defined as a central neurological deficit after surgery and was confirmed by computed tomography (CT) or magnetic resonance imaging. Death occurring within the hospital was defined as *hospital mortality*.

We defined the location of the primary entry using the intraoperative findings. The entry sites were classified as follows: the *ascending aorta* was defined as the location extending up to the bifurcation of the brachiocephalic artery. The *proximal arch* extended from the bifurcation of the brachiocephalic artery to the bifurcation of the left common carotid artery, and the distal arch from the bifurcation of the left common carotid artery to the bifurcation of the left subclavian artery. The *descending aorta* was defined as the location beyond the bifurcation of the left subclavian artery. Additionally, if the entry could not be identified from the intraoperative findings, it was set as unknown.

At the time of discharge, the activity of daily living (ADL) status was evaluated and was classified according to the Japan National Clinical Database as “severely compromised,” “moderately compromised,” or “not affected,” corresponding to Modified Rankin Scale [13] grades 5, 4, and 0–3, respectively.

Postoperative false lumen patency rate was evaluated within 6 months after surgery using contrast-enhanced CT and classified as “fully thrombosed,” “thoracic aorta patent,” “thoracic and abdominal aorta patent,” “abdominal aorta patent,” and “unknown.”

### Statistical analysis

All statistical analyses were performed using the statistical software EZR (Easy R) on the R commander [14]. Continuous variables were expressed as mean values ± standard deviation and compared using Student’s *t* test, whereas categorical variables were expressed as counts and percentages and compared using the *χ*^2^ test. Hospital mortality was evaluated using multivariate Cox proportional hazards regression analysis. Kaplan–Meier survival curves were constructed to assess differences in survival between the older and below-80 patient groups and between the surgical and conservative treatment groups.

Lastly, the survival distributions were compared using log-rank tests. Statistical significance was set at *p* < 0.05.

## Results

The preoperative patient characteristics are summarized in Table 1. DeBakey type II aortic dissection was significantly more common in the older group, whereas type I was significantly more common in the below-80 group. Complete thrombosis of the false lumen was significantly more common in the older group. However, the 30-day operative mortality of the Japan score did not differ between the two groups.

**Table 1:** Preoperative characteristics

	Elderly group (*n* = 31)	Non-elderly group (*n* = 143)	*p* value
Age	83.6 ± 2.8	64.0 ± 10.4	< 0.001
Male sex	10 (32.2)	74 (51.7)	0.073
Hypertension	19 (61.2)	88 (61.5)	0.979
Hemodialysis	0	2 (1.3)	0.507
Coronary artery disease	0	4 (2.7)	0.346
Cerebrovascular disease	2 (6.4)	14 (9.7)	0.559
Dissection related status			
DeBakey			
I	16 (51.6)	108 (75.5)	0.007
II	15 (48.4)	32 (22.3)	0.003
III	0	3 (2.1)	0.415
Fully thrombosed false lumen	14 (45.1)	33 (23.0)	0.012
Preoperative shock	8 (25.8)	36 (25.1)	0.941
Tracheal intubation	2 (6.4)	15 (10.4)	0.492
Cardiac tamponade	9 (29.0)	33 (23.0)	0.482
Pericardial drainage	8 (25.8)	20 (13.9)	0.104
Organ malperfusion	6 (19.3)	48 (33.5)	0.121
CNS	6 (19.3)	37 (25.8)	0.445
Transient	1 (3.2)	15 (10.4)	0.204
Persistent	5 (16.1)	22 (15.3)	0.917
Japan score			
30 days operative mortality	9.6 ± 6.7	10.9 ± 13.7	0.296

CNS, central nervous system

The intra-operative data are summarized in Table 2. Primary entry was found in the ascending aorta in 51.6% and 32.8% of the older and below-80 groups, respectively (*p* = 0.049); however, the primary entry resection rates did not differ between the two groups (87.0% vs. 82.5%, *p* = 0.535). Ascending or hemiarch replacement was performed in all patients in the older group, but only in 57.3% of patients in the below-80 group (*p* < 0.001). The operative, CPB, and selective cerebral perfusion times were significantly longer in the below-80 group than in the older group; the intraoperative blood loss was significantly lower in the older group than in the below-80 group (*p* = 0.023).

**Table 2:** Intra-operative data

	Elderly group (*n* = 31)	Non-elderly group (*n* = 143)	*p* value
Entry site			
Ascending aorta	16 (51.6)	47 (32.8)	0.049
Proximal arch	8 (25.8)	33 (23.0)	0.745
Distal arch	5 (16.1)	40 (27.9)	0.172
Descending aorta	0	10 (6.9)	0.129
Unknown	2 (6.4)	13 (9.0)	0.635
Entry resection	27 (87.0)	118 (82.5)	0.535
Procedures			
Ascending/Hemiarch replacement	31 (100)	82 (57.3)	< 0.001
Total arch replacement	0	61 (42.7)	< 0.001
Concomitant procedures			
AVR	0	4	
CABG	2	7	
Root replacement	1	3	
Operative data			
Duration, minutes			
Operation	347.1 ± 92.2	425.1 ± 123.6	< 0.001
Cardiopulmonary bypass	206.8 ± 52.0	245.2 ± 69.3	0.002
Circulatory arrest	58.0 ± 9.4	55.2 ± 14.5	0.151
Cardiac arrest	153.7 ± 33.0	160.5 ± 39.1	0.184
Selective cerebral perfusion	57.5 ± 16.4	99.8 ± 58.5	< 0.001
Blood loss, mL	1489.7 ± 686.4	2046.7 ± 1517.5	0.023

AVR, aortic valve replacement; CABG, coronary artery bypass grafting

The postoperative data are summarized in Table 3. Stroke was found in 25.8% and 27.2% of the older and below-80 group patients, respectively. There were five in-hospital deaths in the older group (mortality, 16.1%). The causes of death were stroke (*n* = 3), sepsis (*n* =1), and rupture of the residual aorta (*n* = 1).

**Table 3:** Postoperative data

	Elderly group (*n* = 31)	Non-elderly group (*n* = 143)	*p* value
Mechanical ventilation time ≧ 48 h	9 (25.0)	35 (24.4)	0.596
Renal replacement therapy	3 (9.6)	10 (6.9)	0.606
Stroke	8 (25.8)	39 (27.2)	0.867
Length of ICU stay, days	8.0 ± 8.6	6.5 ± 6.6	0.170
Length of hospital stay, days	25.3 ± 23.3	27.2 ± 22.1	0.332
ADLs status at discharge			
Not affected	12 (38.7)	97 (67.8)	0.002
Moderately compromised	8 (25.8)	27 (18.8)	0.383
Severely compromised	6 (19.3)	8 (5.5)	0.010
Discharge to home	13 (41.9)	91 (63.6)	0.025
Hospital death	5 (16.1)	11 (7.6)	0.140
Postoperative false lumen patency			
Fully thrombosed	16 (51.6)	43 (30.0)	0.021
Thoracic aorta patent	2 (6.4)	5 (3.4)	0.447
Thoracic and abdominal aorta patent	4 (12.9)	67 (46.8)	*p* < 0.001
Abdominal aorta patent	1 (3.2)	13 (9.0)	0.276
Unknown	8 (25.8)	15 (10.4)	0.022
Late aortic events			
TEVAR	1	5	
Descending aorta rupture	1	1	
Descending aorta replacement		2	
Anastomotic pseudoaneurysm		2	
Others		2	

ICU, intensive care unit; ADLs, activities of daily living; TEVAR, thoracic endovascular aortic repair

There were 11 in-hospital deaths in the below-80 group (mortality, 7.6%). The causes of death were stroke (*n* = 8), postoperative bleeding (*n* = 2), and rupture of the residual aorta (*n* = 1). The discharge rates were 41.9% and 63.6% in the older and below-80 groups, respectively (*p* = 0.025). Regarding the ADL status at discharge, non-affected patients were significantly more common in the below-80 group (*p* = 0.002), and the severely compromised patients were significantly more common in the older group (*p* = 0.010). Regarding postoperative false lumen patency, complete thrombosis of the false lumen was significantly more common in the older group (*p* = 0.021), whereas thoracic and abdominal aorta patency of the false lumen was significantly more common in the below-80 group (*p* < 0.001). The late aortic events were identified, including thoracic endovascular aortic repair (TEVAR) descending aorta rupture, descending aorta replacement, and anastomotic pseudoaneurysm (Table 3).

Surgical and conservative treatment sub-groups of the older group were compared and data are shown in Table 4. Forty-five patients in the older group chose to recieve conservative therapy because of comorbidities (decreased ADL [12 cases], thrombosis and reduction of the false lumen [7 cases], and advanced dementia [5 cases]) or refusal of surgical treatment by patients or their families (21 cases). Dementia and decreased ADL (inability to walk without assistance) cases were significantly higher in the conservative treatment group. However, the length of hospital stay was significantly shorter in the conservative treatment group. The ADL status at discharge and the dissection-related status did not significantly differ between the two treatment groups.

**Table 4:** Characteristics of surgical group and conservative group in elderly patients

	All patients over 80 years of age		
	Surgical group (*n* = 31)	Conservative group (*n* = 45)	*p* value
Age	83.6 ± 2.8	85.4 ± 3.5	0.013
Male sex	10 (32.2)	14 (31.1)	0.915
Preoperative status			
Dementia	1 (3.2)	10 (22.2)	0.020
Cerebrovascular disease	2 (6.4)	4 (8.8)	0.698
ADLs decline	0	13 (28.8)	0.001
Dissection related status			
DeBakey			
I	16 (51.6)	28 (62.2)	0.357
II	15 (48.4)	14 (31.1)	0.127
III	0	1 (2.2)	0.403
Unknown	0	2 (4.4)	0.234
Fully thrombosed false lumen	14 (45.1)	23 (51.1)	0.610
Pericardial drainage	8 (25.8)	7 (15.5)	0.269
ADLs status at discharge			
Not affected	12 (38.7)	11 (24.4)	0.183
Moderately compromised	8 (25.8)	13 (28.8)	0.767
Severely compromised	6 (19.3)	7 (15.5)	0.665
Hospital death	5 (16.1)	14 (31.1)	0.138
Length of hospital stay, days	25.3 ± 23.3	14.9 ± 12.0	0.006
Discharge home	13 (41.9)	11 (24.4)	0.106

ADLs, activities of daily living

### Hospital mortality

Multivariate logistic regression analysis identified significant risk factors associated with hospital mortality, namely age, preoperative intubation, CPB time, and postoperative stroke (Table 5).

**Table 5:** Multivariate logistic regression analysis for risk factors associated with hospital mortality

Variable	Odds ratio	95% Confidence interval	*p* value
Age	9.38	1.64–53.6	0.01
Malperfusion	1.07	0.28–4.09	0.91
CNS (transient)	0.52	0.06–4.34	0.55
CNS (persistent)	0.67	0.13–3.54	0.64
Preoperative intubation	14.0	2.47–79.0	*p* < 0.01
CPB long (≧ 240 min)	6.40	1.12–36.5	0.03
TAR	1.98	0.32–12.0	0.45
Postoperative stroke	10.8	2.62–44.6	*p* < 0.01

CNS, central nervous system; CPB, cardiopulmonary bypass; TAR, total arch replacement

### Long-term mortality

**Fig. 1:**
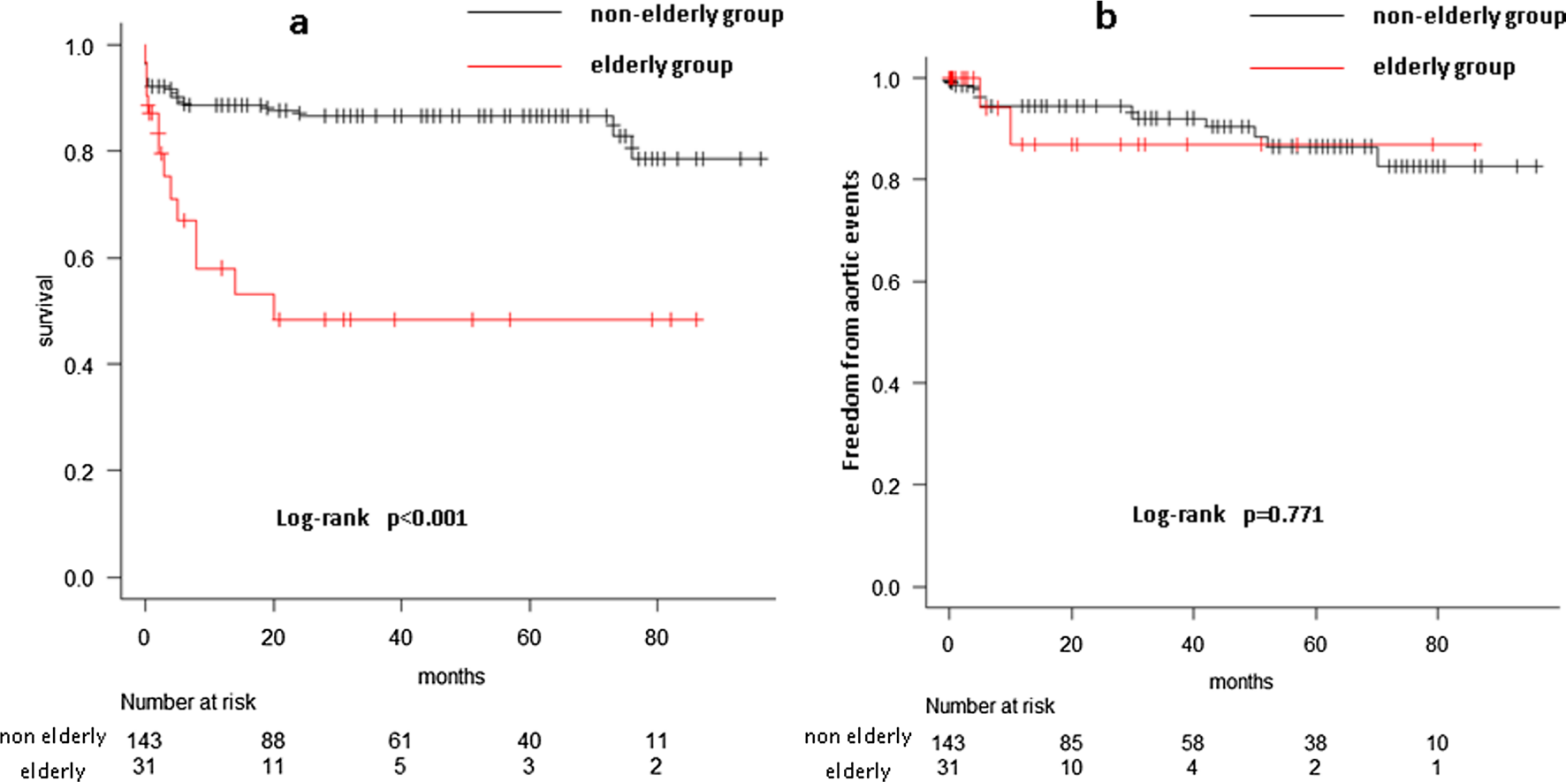
**a** The 5-year survival rate was significantly lower in the older group (48.4% ± 10.3%) than the below-80 group (86.7% ± 2.9%). **b** The rate of freedom from aortic events at 5 years did not significantly differ between the older (86.9% ± 8.7%) and below-80 group (86.5% ± 3.9%) groups

**Fig. 2:**
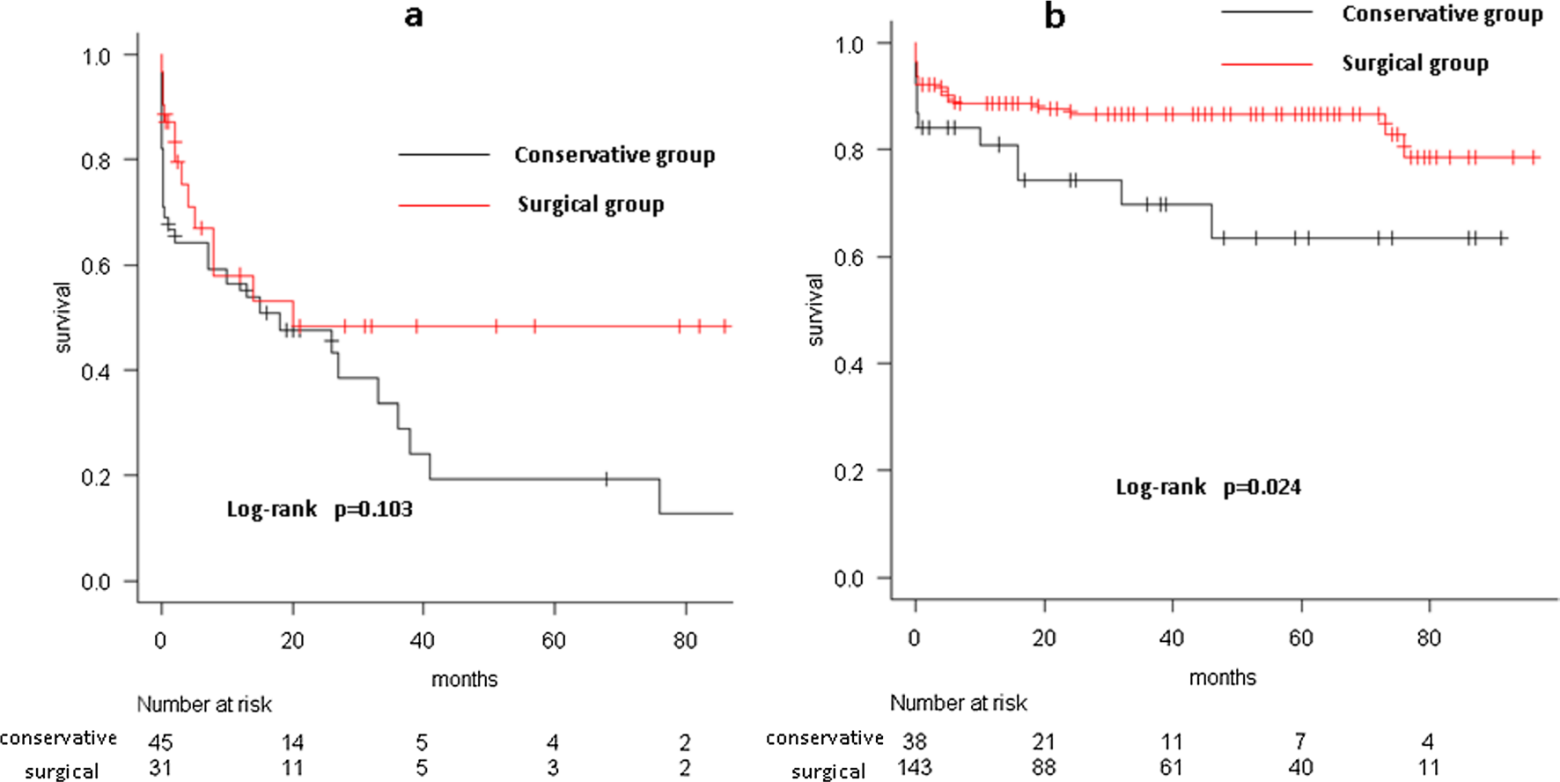
**a** In the older group, the 5-year survival rate did not significantly differ between the surgical (48.4% ± 10.3%) and conservative (19.2% ± 8.0%) treatment groups. **b** In the below-80 group, the 5-year survival rate in the surgical treatment group (86.7% ± 2.9%) was significantly higher than that of the conservative treatment group (63.5% ± 9.6%)

The overall mean follow-up period was 30.3 ± 28.3 months (median, 21 months; range, 0.03–93 months). The mean follow-up period was 19.3 ± 25.6 months (median, 6 months; range, 0.03–86 months) in the older surgical group, 17.0 ± 23.4 months (median, 10 months; range, 0.03–93 months) in the older conservative group, 36.8 ± 27.9 months (median 32 months, range 0.03–93 months) in the below-80 surgical group, and 30.8 ± 28.7 months (median, 24 months; range, 0.03–91 months) in the below-80 conservative group. The 5-year survival rates were 48.4% ±10.3% and 86.7% ± 2.9% in the older and below-80 groups, respectively (*p* < 0.001, Fig. 1a). The rates of freedom from aortic events at 5 years were 86.9% ± 8.7% and 86.5% ± 3.9% in the older and below-80 groups, respectively (*p* = 0.771, Fig. 1b). The 5-year survival rate of the conservative treatment sub-group was 19.2% ± 8.0% in the older group, which was not significantly different from that of the surgical treatment subgroup (*p* = 0.103, Fig. 2a). Similarly, we compared the 5-year survival rates between the surgical (86.7% ± 2.9%) and conservative (63.5% ± 9.6%) sub-groups in the below-80 group; the prognosis was significantly better in the surgical subgroup (*p* = 0.024, Fig. 2b).

## Discussion

Japan has one of the highest life expectancy rates worldwide and the likelihood of an acute type A aortic dissection in older patients is particularly high in the aging society. Acute type A aortic dissection remains a fatal disease despite recent improvements in surgical outcomes and the patients experience various post-surgical complications. Based on several reports on surgical outcomes in older patients with acute type A aortic dissection, the surgical mortality rate is 3.7–35% [2–4, 9, 10, 15–18]. TAR has higher mortality and morbidity rates than ascending or hemiarch replacement [19]. Another study reported that despite longer CPB, aortic clamp, and circulatory arrest times, there was no difference in mortality and morbidity rates between hemiarch replacement and TAR [20]. Some studies reported that the surgical procedure in all older patients with acute type A aortic dissection was either ascending or hemiarch replacement [2, 9], whereas others reported ascending or hemiarch replacement in 90% of patients [3, 10, 17, 19]. These reports indicate that older patients with acute type A aortic dissection avoid TAR as a surgical procedure. We selected ascending or hemiarch replacement as the surgical procedure for acute type A aortic dissection in patients > 80 years of age, regardless of the primary entry site. It is important to evaluate whether the inability to resect the primary entry affects the prognosis because non-resection of the primary entry is a predictor of survival and distal aortic events? [21]. Furthermore, aggressive primary entry resection can enhance false lumen thrombosis and reduce aortic reoperation [22].

In contrast, a previous study suggests that aortic events do not change even if the entry is not resected and the patient is followed up carefully [23]. In our study, all older group patients had selected ascending or hemiarch replacement as the surgical procedure. The entry resection rate in the older group was 87%; however, the entry resection rate and long-term aortic events did not differ between the older and below-80 group patients. Descending aorta replacement, TEVAR, and rupture of the descending aorta as late aortic events occurred in two cases in the older group and eight cases in the below-80 group (Table 3); both cases in the older group and five cases in the below-80 group were able to undergo entry resection. Therefore, even if the entry can be resected, aortic remodeling may proceed if there is a reentry in the residual aorta and careful follow-up is required. Our multivariate analysis identified advanced age and longer CPB time as risk factors of postoperative hospital mortality. The CPB time can be shortened by selecting an ascending or hemiarch replacement for older patients.

Selecting ascending or hemiarch replacement without sticking to the entry resection and shortening the operative time may contribute to the surgical outcomes in the older patients with acute type A aortic dissection; the location of the primary entry tear significantly influences early outcomes and short-and long-term survival of patients [24]. In general, intimal tears are frequently found in the segments exposed to the greatest shear stress, namely the ascending aorta’s right lateral wall (opposite the main pulmonary artery) or descending aorta’s proximal segment [25]. In our study, the primary entry was likely to occur in the ascending aorta, and DeBakey type II dissection was likely to occur in older patients. There are other reports such as this [3, 4]. Older patients may be more likely to be stressed by the ascending aorta due to the prognosis of arteriosclerosis. Therefore, primary entry is common in the ascending aorta of older patients.

Moreover, the indications for surgery and the postoperative course in older patients showed that although general condition before surgery affects the outcome, advanced age alone should not be considered a contraindication to acute type A aortic dissection repair [2, 3, 6]. An age of ≥ 80 years was a risk factor for in-hospital mortality during operation for acute type A aortic dissection [4]. Hata and colleagues [9] described that older patients had post-surgical complications, such as cerebral damage, depression, pneumonia, or renal failure and ultimately became bedridden, causing significant mental, physical, and economic stress. Aoyama and colleagues [19] reported that the discharge rate was significantly higher in the conservative treatment group (52.8%) than in the surgical treatment group (42.8%, *p* < 0.01) of older (≥ 80 years) patients with acute type A aortic dissection.

Furthermore, the duration of hospital and intensive care unit stay was significantly longer, and medical expenses were significantly higher in the surgical treatment group than in the conservative treatment group. In our study, the older group had a significantly worse ADL status at discharge and a significantly lower discharge rate (41.9%) than the below-80 years group (63.6%).

Moreover, 12 patients in the older group were transferred to the rehabilitation hospital after surgery, and four of them died due to pneumonia or heart failure within 1 year after surgery.

The 5-year survival rates did not differ between the surgical and conservative treatment sub-groups of the older group; however, even if the life of the older patients can be saved through surgical treatment, it may lead to a decrease in quality of life, and based on the long-term prognosis, the patient may not benefit from surgical treatment. Therefore, surgical indications should be carefully considered for older patients with acute type A aortic dissection. Older patients are likely to experience irreversible physical deterioration after surgery and may progress to dementia or be bedridden, thus increasing burden and stress on the family. Post-intensive care syndrome [26, 27] should be considered, and cardiac surgeons should work together for the patient’s postoperative rehabilitation to maximize the possibility of discharging the patient.

In this study, postoperative stroke was more frequent in both the older and below-80 groups. Four of eight postoperative stroke cases in the older group and 14 of 39 postoperative stroke cases in the below-80 group had persistent central nervous system malperfusion before surgery. Therefore, postoperative stroke cases may include preoperative stroke cases. In our study, stroke was the most common cause of postoperative death in both the older and below-80 groups and a major risk factor affecting hospital mortality. Therefore, stroke reduction is the most important factor for improving surgical results. Effective axillary artery cannulation could prevent stroke after aortic arch replacement [28–30]. In addition, we are currently trying to actively introduce and improve cannulation of the right axillary artery in CPB to reduce postoperative neurological damage. Furthermore, we have recently collected data to assess pre-and postoperative ADL using the Barthel index, which may help predict postoperative outcomes in older patients in our subsequent study.

The present study was limited by its retrospective, single-center design. Furthermore, the small number of cases makes it difficult to draw a clear conclusion. For instance, the hospital mortality and long-term survival did not significantly differ between the surgical and conservative treatment subgroups of the older group. In the future, as the number of cases increases, it may be possible to obtain results showing that hospital death is significantly reduced in the surgical treatment group. Therefore, the superiority of surgical treatment for acute type A aortic dissection may increase, even in older patients.

## Conclusion

In older patients, primary entry due to acute type A aortic dissection is likely to occur in the ascending aorta. Furthermore, DeBakey type II dissection is significantly more common in older patients than in patients below 80 years of age, which is consistent with the selection of ascending or hemiarch replacement as the surgical procedure. Older patients are prone to irreversible physical deterioration after surgery; therefore, it may not be possible to take advantage of the surgical treatment. Moreover, surgical treatment could not achieve a significant survival advantage over the conservative approach; thus, it may not always be the reasonable treatment of choice for older patients. This point should be explained to the patient’s family while deciding on whether to adopt the surgical approach.

## Data Availability

Please contact the corresponding author regarding any data requests.

## Abbreviations

TAR: Total arch replacement
ET: Elephant trunk
FET: Frozen elephant trunk
CT: Computed tomography
AVR: Aortic valve replacement
CABG: Coronary artery bypass grafting
CPB: Cardiopulmonary bypass
ADLs: Activities of daily living
TEVAR: Thoracic endovascular aortic repair
